# Supplementary material for: Independent replications reveal anterior and posterior cingulate cortex activation underlying state anxiety-attenuated face encoding
Source: Commun Psychol. 2024 Aug 24;2:80. doi: 10.1038/s44271-024-00128-y (PMC11343718; doi:10.1038/s44271-024-00128-y)
Supplement: Supplementary file 2 — Supplementary Material [file 44271_2024_128_MOESM2_ESM.pdf]

Supplementary Material

Threat-of-Shock Face Recognition Task: Internal Consistency

Since the 36 trials were aggregated to compute the mean face recognition accuracy at retrieval for each of the four task blocks, per subject, we checked Cronbach's Alpha as a measure of internal consistency for the trial-level data. We report the alpha for each condition separately and confirm that the internal consistency ranges from acceptable (0.7-0.8) to good (0.8-0.9):

1. Block with safety at encoding followed by safety at retrieval: 0.831
2. Block with safety at encoding followed by threat-of-shock at retrieval: 0.795
3. Block with threat-of-shock at encoding followed by safety at retrieval: 0.729
4. Block with threat-of-shock at encoding followed by threat-of-shock at retrieval: 0.791

Behavioural Results when encoding faces under threat-of-shock compared to safety: transformed data.

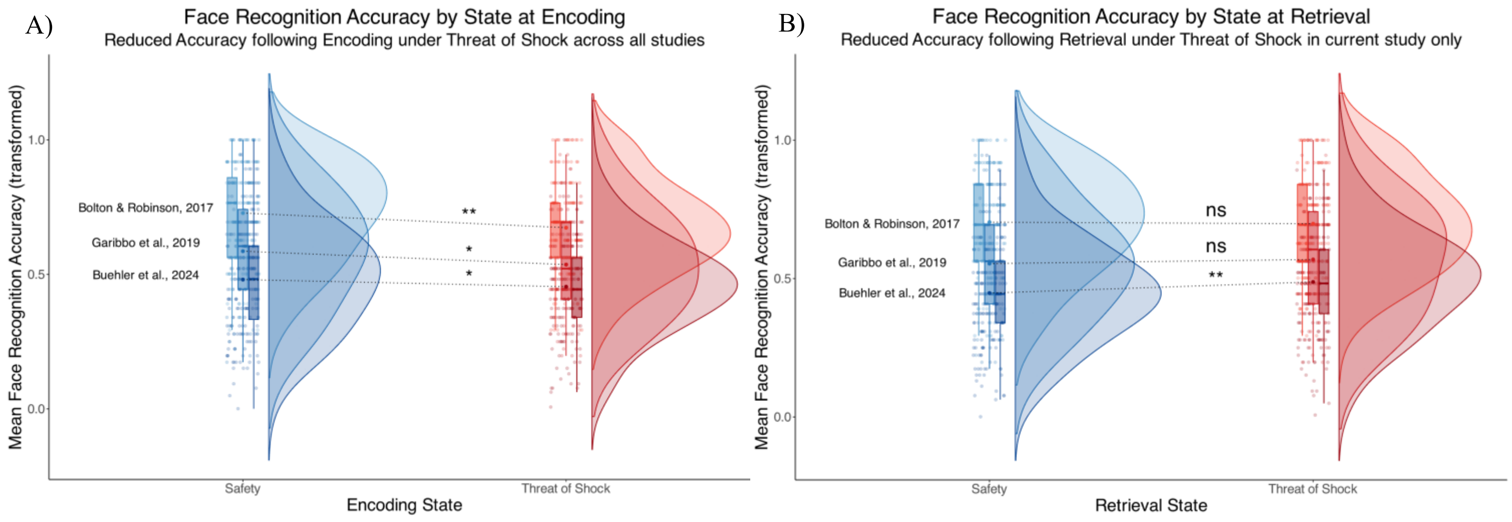

Supplementary Figure 1. Transformed behavioural results *combining the current study (Buehler et al. 2024: N=92) with behavioural data from two previous studies (Bolton & Robinson, 2017: N=86; Garibbo et al., 2019: N=32) reveal that face recognition accuracy is affected by the state, comparing threat-of shock (red) to safety (blue), during A) encoding but not B) retrieval.*

Exploratory Summary Statistic Meta-Analysis of Behaviour across studies

In addition to the mega-analysis, we report the results of a standard meta-analysis, which utilizes the summary statistics and a weighting mechanism to account for differences in precision between different samples (Eisenhauer, 2021). We utilized the multivariate random effects meta-analysis model fit using the default rma.mv function from the metafor package in R (Viechtbauer, 2010) with the following syntax in R: *rma.mv(yi=d, V= Var(d), slab=study, data, random=~1|study).* In this model the individual study estimates were weighted using the inverse sampling variance of the study relative to the estimated amount of heterogeneity across all studies. Due to the within-subjects design, we calculated the Cohen's d effect size (*d*) and its variance (*Var(d))* from the ANOVA F-statistics and sample sizes as follows: $d = \sqrt{\frac{F}{N}}$, $Var(d) = \frac{1}{N} + \frac{d^2}{2N}$.

The meta-analysis revealed that a significant difference in face recognition accuracy between the threat-of-shock and safe control condition was only evident for encoding (z=4.19, p<0.001, *mean difference*=0.30, $CI_{95\%}$=[0.16, 0.43], *see supplementary figure 2a*), but not retrieval (z=1.75, *p*=0.08, *mean difference*=0.18, $CI_{95\%}$=[-0.02, 0.38], *see supplementary figure 2b*).

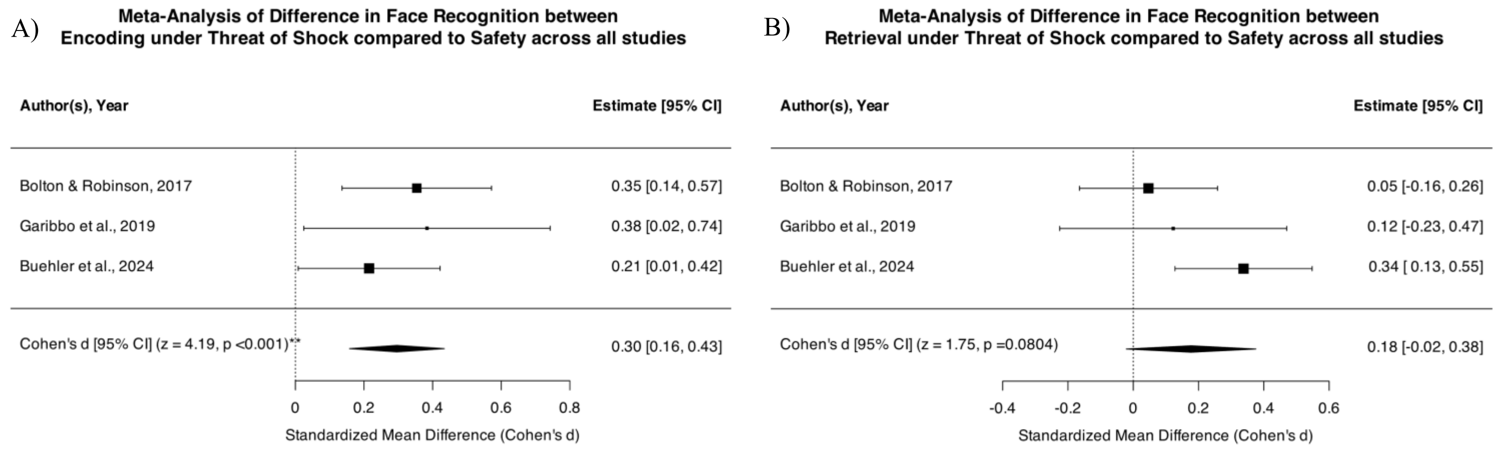

Supplementary Figure 2. Summary Statistic Meta-Analyses *were conducted on the main effects across the current study sample (Buehler et al. 2024: n=92) and the two previous studies (Bolton & Robinson, 2017: n=86; Garibbo et al., 2019: n=32) utilizing the same task paradigm. These showed that at the meta-analytic level a difference in face recognition accuracy is evident when A) encoding occurred under threat-of-shock compared to safety but not for B) retrieval under threat-of-shock compared to safety.*

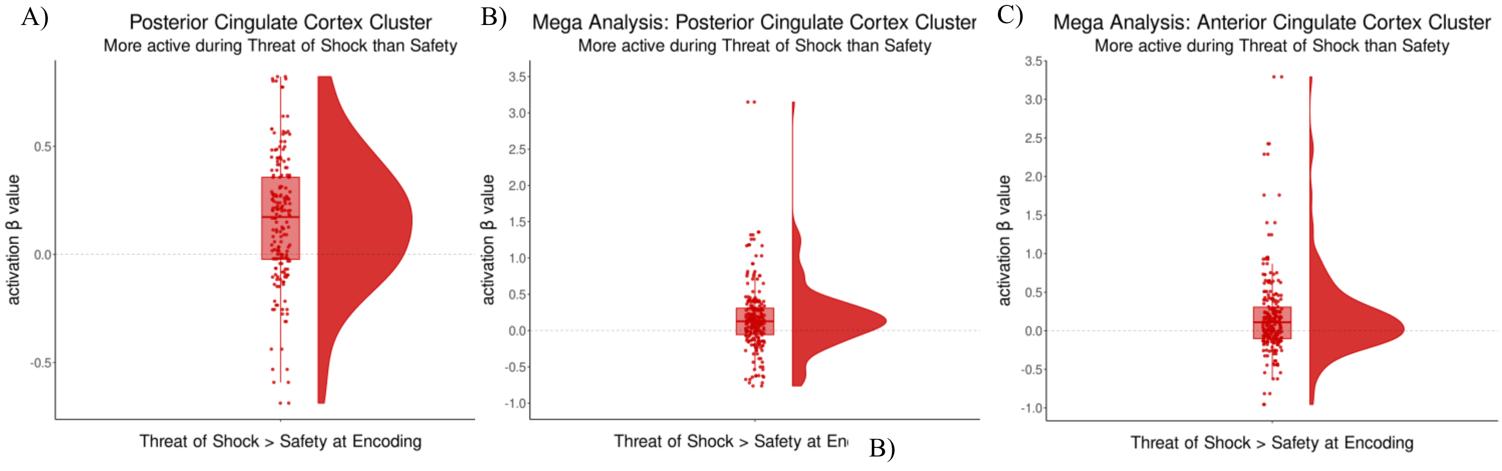

Supplementary Figure 3. Beta coefficients extracted for the significant model contrasts from the whole-brain analysis of neural activation *showing that in the current study A) there was a significant effect in the posterior cingulate cortex cluster (PCC) encoding of faces under threat-of-shock compared to safety. Further, when combining the current study and within-subject results from Garibbo et al. (2019) in a whole brain mega-analysis, there was a significant effect while encoding faces under threat-of-shock compared to safety in both B) the posterior cingulate cortex (PCC) and C) the anterior cingulate cortex (ACC). Note the beta coefficients were extracted from the significant whole-brain group-level contrasts shown in the main results figure 2 and plotted here for visualisation purposes only.*

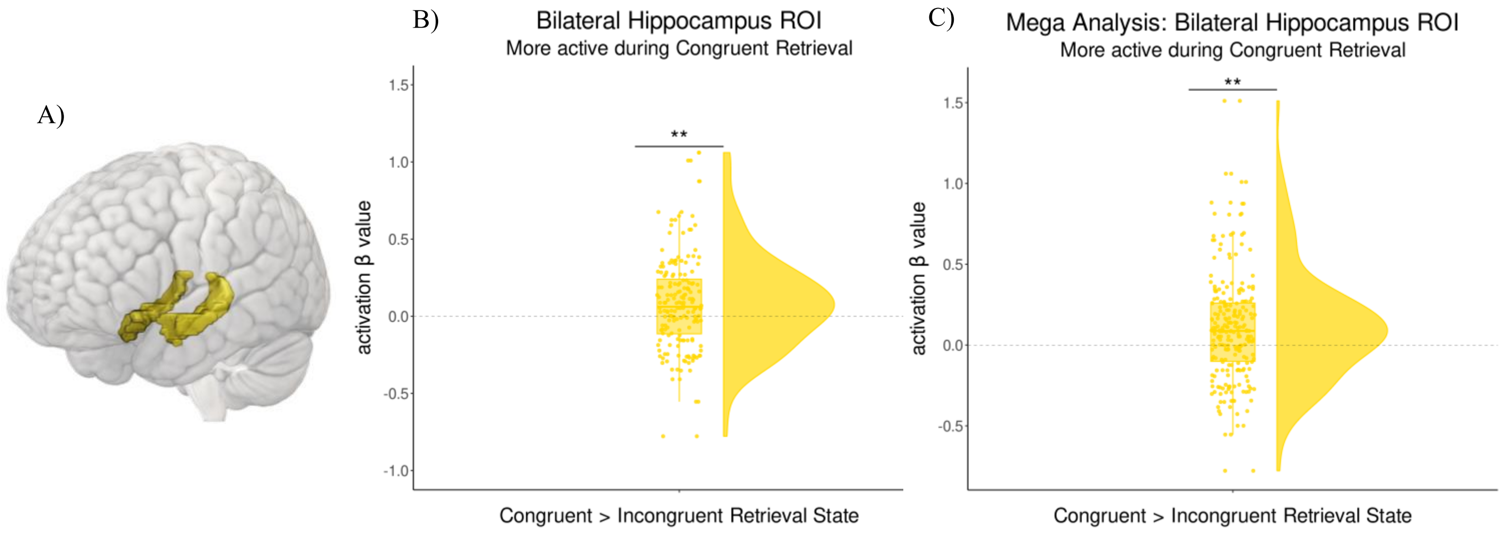

Supplementary figure 4. Beta coefficients for the state-congruency contrast *extracted from the significant A) bilateral hippocampus ROI and showing increased activation in the hippocampus when the state during retrieval is congruent compared to incongruent with encoding in B) the current study (Buehler et al. 2024: n=92) and C) a mega-analysis combining the current study with Garibbo et al. (2019).*

Neural Activation when encoding faces under threat-of-shock compared to safety: additional cluster in whole-brain analysis.

Primary Analysis: Another smaller cluster emerged in the right angular gyrus, but it passed cluster-correction only close to the minimum required threshold of 23.5 (size: 27, voxels, peak: x=-55.5, y=+52.5., z=+31.5, centre of mass: x=-55.2, y=+52.4., z=+30.9)

Exploratory Behavioural Hypothesis: Improved recognition of faces retrieved in the same state (threat-of-shock or safety) as they were encoded in.

At the behavioural level, there was no significant effect of state congruency in the previous studies (see Bolton & Robinson, 2017: N=86; Garibbo et al., 2019: N=32). There was only no convincingly significant difference in our sample (‘Buehler et al. 2024’: N=92, F=0.482, df=91, p=0.489, η_p^2=0.005, see supplementary figure 2a) between state congruent (*Mean=0.669, SD=0.158*) and incongruent (*Mean=0.665, SD=0.142*) retrieval. We also find no differences based on state congruency when combining all three studies in a meta-analysis (z=1.52, p=0.128, *Mean difference*=0.11, $CI_{95\%}$=[-0.03, 0.24], see supplementary figure 2a) or individual participant data (IPD) mega-analysis (F=0.024, df=207, p=0.878, η_p^2=0.000).

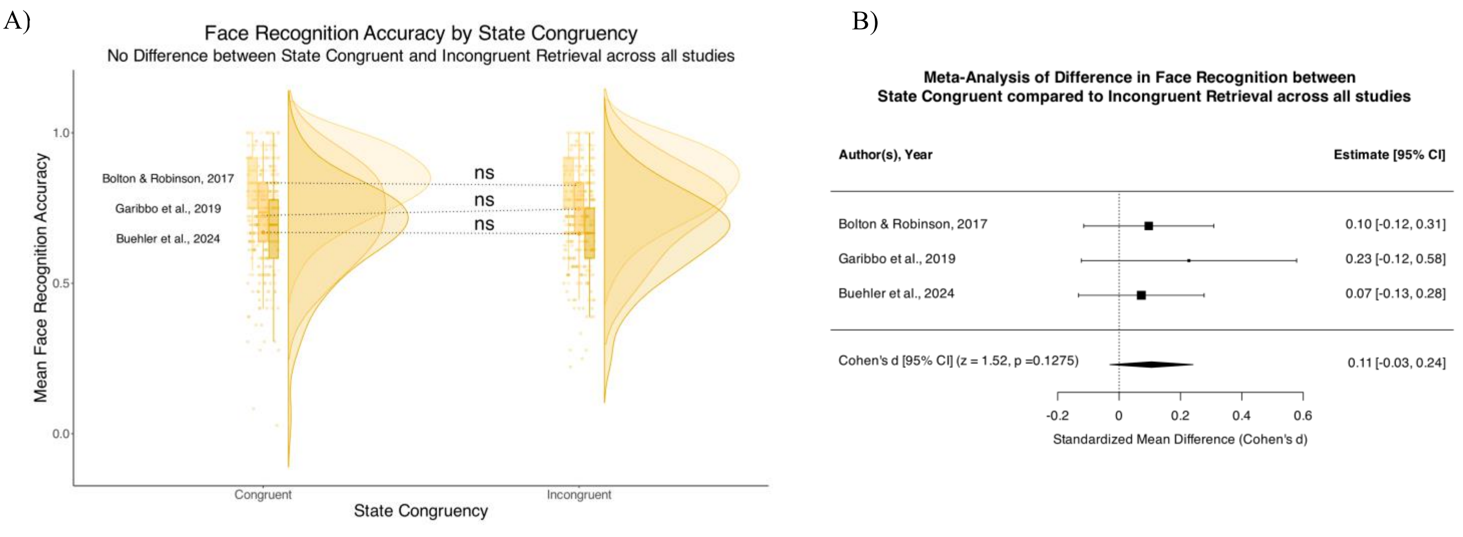

Supplementary Figure 5. State-Congruency *(threat-of-shock or safety) has no statistically significant behavioural effect in A) the current study (Buehler et al. 2024: N=92) or B) when combined in a meta-analysis with two previous studies (Bolton & Robinson, 2017: N=86; Garibbo et al., 2019: N=32).*

Exploratory Behavioural Hypothesis: State congruency improves reaction time at retrieval.

An additional exploratory within-subject ANOVA was run to investigate the effect of state congruency (threat-of-shock/safety at encoding followed by threat-of shock/safety at retrieval) compared to incongruency (threat-of-shock/safety at encoding followed by safety/threat-of shock at retrieval) on face recognition reaction time at retrieval.

We found no difference in reaction time between state congruent and incongruent retrieval in our sample (F=0.018, df=91, p =0.895, η_p^2=0.000).

A)

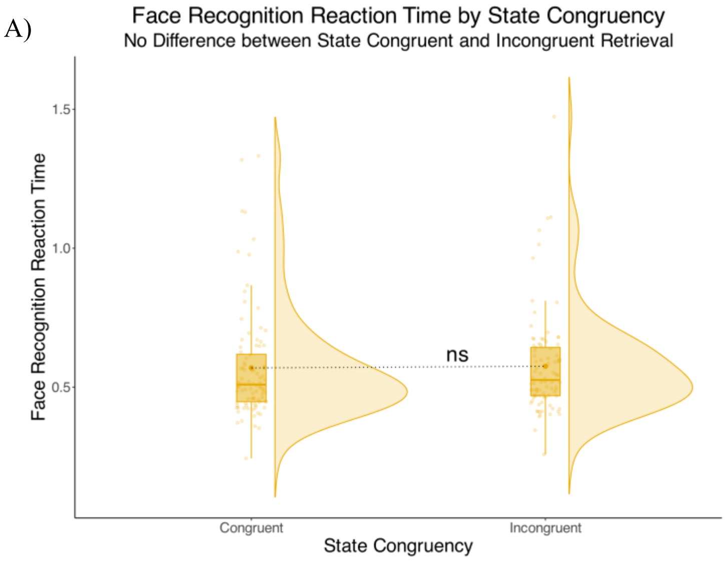

Supplementary Figure 6. State Congruency *A) does not affect reaction time behaviour on the face recognition task.*

Exploratory Behavioural Hypothesis: Threat-of-Shock Retrieval increases incorrect classification of faces as familiar.

Some evidence in the literature suggests that high arousal states might be associated with heightened feelings of (false) familiarity and bias individuals to report that they recognize a face (Fiacconi et al., 2016). We utilized signal detection theory to formalize a post-hoc exploratory hypothesis that participants might be more likely to incorrectly classify faces as seen before (i.e., false familiarity) when retrieval occurs under threat-of-shock compared to safety. This could be reflected in a significantly more negative decision criterion when retrieval occurs under threat-of-shock compared to safety.

Using the signal detection theory framework, behavioural responses on the threat-of-shock potentiated face recognition task were categorizes as hits, misses, false alarms and correct rejections (see table X). Subsequently the proportion of each of these categories of response is computed for every individual to obtain a hit rate ($\frac{sum\ of\ hits}{sum\ of\ hits+sum\ of\ misses}$), false alarm rate ($\frac{sum\ of\ false\ alarms}{sum\ of false\ alarms+sum\ of\ correct\ rejections}$), miss rate ($\frac{sum\ of\ misses}{sum\ of\ hits+sum\ of\ misses}$), correct rejection rate ($\frac{sum\ of\ false\ alarms}{sum\ of false\ alarms+sum\ of\ correct\ rejections}$). The hit rate and false alarm rate can be further used to obtain a measure of participant's perceptual sensitivity (d') in terms of distinguishing between signal absent and signal present trials, which is defined as the difference between the inverse normal distribution of the hit rate and the false alarm rate:

Computed in R as d'*=qnorm(hit rate)-qnorm(false alarm rate)*

A larger d' therefore denotes a higher sensitivity (i.e., distance between the signal present and signal absent distributions). In addition, the participant's decision criterion (c) can be computed to quantify their perceptual bias (i.e., propensity to respond yes versus no). This is defined as the negative value of half of the sum of the inverse normal distribution of the hit rate and the false alarm rate:

Computed in R as *c=-0.5*(qnorm(hit rate)+qnorm(false alarm rate))*

This describes the distance between the participant's threshold for giving a yes versus no response and the midpoint of the signal absent and signal present distributions. With higher false alarm rates than the miss rates, *c* becomes increasingly negative and reflects a bias towards responding 'yes' regardless of the signal, while c is increasingly positive as the miss rate exceeds the false alarm rate and responses are biased towards 'no'.

		**Signal**: *Was this face presented before?*	
		**Yes**	**No**
**Response**:	**Yes**	Hit	False Alarm
Was this face seen before?	**No**	Miss	Correct Rejection

Table 1. *Signal Detection Theory: Categories of Responses based on Signal on any trial*

On this task it is possible to get hit and false alarms rate of 0 and 1, in which case no meaningful d' can be computed. In those instances a correction was applied by converting a

proportion of 0 to 1/(2N) and a proportion of 1 to 1−1/(2N), whereby N is the total number of trials for the condition (see Huang & Ferreira, 2020).

There was no statistically significant difference between the false alarm rate (t=-1.877, df=91, p=0.064, 95% CI: -0.049, 0.001) or decision criterion (t=-0.298, df=91, p=0.766, 95% CI: -0.077, 0.057) when retrieval occurred under threat-of shock (*false alarm rate: Mean=0.281, SD=0.175, decision criterion: Mean=0.086, SD=0.482*) compared to safety (*false alarm rate: Mean=0.305, SD=0.172, decision criterion: Mean=0.096, SD=0.506*).

However, it is important to note that while the threat-of-shock potentiated face recognition paradigm is a two-alternative forced choice task there was a time-limit of 2 seconds after which an incorrect response was automatically recorded. This cannot be captured by the signal detection theory framework, meaning its results will be biased by not taking those trials into account and thus need to be interpreted cautiously and only as an exploratory subsidiary to the main analyses.

Exploratory Behavioural and Neural Hypotheses: Reduced recognition and increased ACC activation for fearful compared to non-fearful faces under threat-of-shock.

We further hypothesized that participants would show reduced accuracy in face recognition during retrieval for fearful compared to non-fearful (happy and neutral) faces that were encoded under threat-of-shock compared to safety. This may be because threat-relevant stimuli that signal potential dangers in the environment likely demand more attentional control, particularly in a state of anxiety (Bishop et al., 2004), which may further reduce the ability to accurately encode the faces with fearful expressions.

For the behavioural analysis, we extended our primary within-subject ANOVA of encoding and retrieval state on face recognition to account for face valence, using a binary comparison of fearful (fear) to non-fearful (happy and neutral) faces. Of interest here were only the interaction terms assessing if the effect of face valence on face recognition accuracy differs under threat-of-shock compared to safety (encoding state*emotion, retrieval state*emotion). To explore any difference in neural activation underlying face valence, we used the onsets times for specific valences of face stimuli during threat-of-shock as well as safe encoding and

retrieval as regressors of interest in the within-subject GLM. Then, we conducted the same group-level ROI analysis of the ACC and exploratory whole-brain analysis as described in the main methods section, with the contrasts of interests specified based on the within-subject models as the interaction between state (threat or safety) and face valence (fearful or non-fearful) for encoding and retrieval separately.

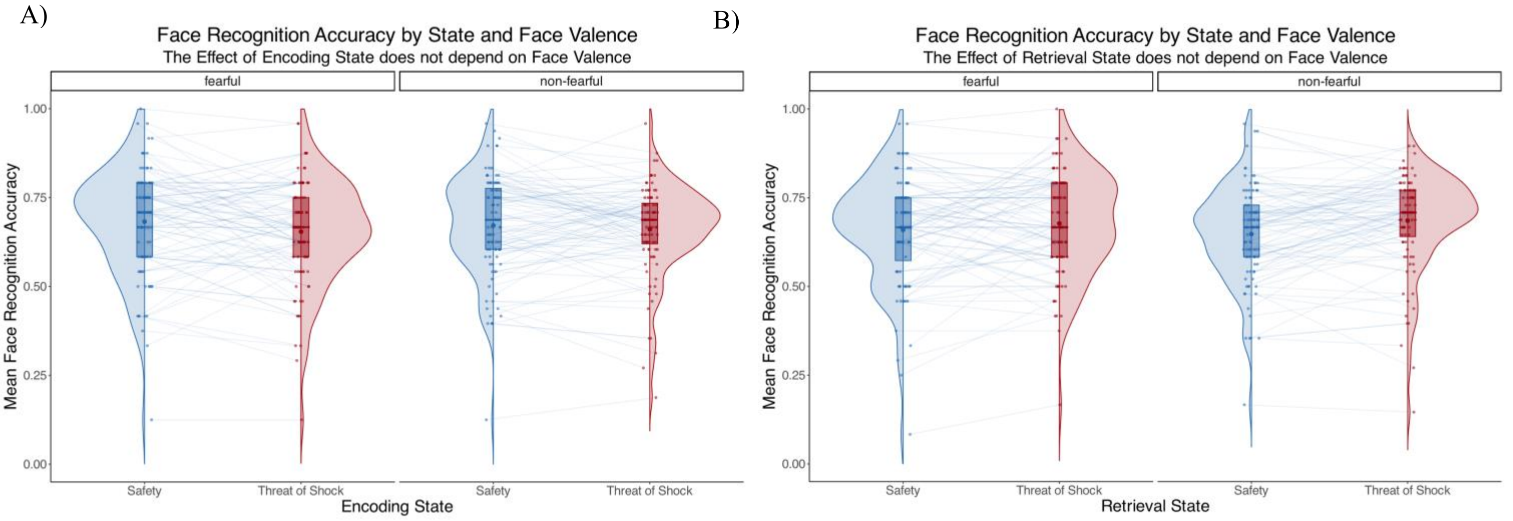

Supplementary Figure 7. Behavioural results *from the current study (N=92) reveal that the effect of state, threat-of-shock or safety, on face recognition accuracy does not interact with the valence of the face stimuli, when comparing fearful to non-fearful (happy and neutral) face expressions during A) encoding or B) retrieval.*

There was no significant interaction between the effect of state (threat-of-shock compared to safety) and the face stimuli valence (fearful compared to non-fearful expressions) on face recognition accuracy during encoding (F=1.280, df=91, p=0.261 η_p^2=0.014) or retrieval (F=1.383, df=91, p=0.243, η_p^2=0.015). We also found no significant neural activation effects for the interaction between state, comparing threat-of-shock to safety, and face valence, comparing fearful to non-fearful expressions, during encoding or retrieval in the anterior cingulate cortex (ACC) ROI or a voxel-wise whole brain analysis (voxel-wise threshold = p<0.001, cluster-level significance threshold of p<0.05).

Exploratory Neural Hypothesis: Increased amygdala-dmPFC connectivity while encoding faces under threat-of-shock.

We further hypothesized increased connectivity of a translational amygdala-prefrontal (i.e., ACC and dorsomedial prefrontal cortex (dmPFC)) circuitry (Etkin et al., 2011; Sierra-Mercado et al., 2011; Vidal-Gonzalez et al., 2006) while encoding faces under threat-of-shock compared to safety. The amygdala may, amongst other things, relay low-level information such as stimulus salience to cortical areas such as the ACC and dmFC (Pessoa, 2009). Previous fMRI studies have also demonstrated that the functional connectivity between these regions is significantly increased in anxiety (Mechias et al., 2010; Robinson et al., 2012, 2014) and appears to be a more reliable within-subject measure than neural activation, particularly across repeated sessions (Nord et al., 2019). But at present, it is unknown if and how this functional circuitry is associated with threat-of-shock mediated face recognition alterations.

For this purpose, we conducted a generalized psychophysiological interaction (gPPI) analysis (Cisler et al., 2014; McLaren et al., 2012), using the resampled (using AFNI's 3dmaskave) left and right amygdala masks from PickAtlas as well as a bilateral sgACC mask from a previous study (Nord et al., 2019) as seed regions. To make group-level inferences, we conducted both ROI and whole-brain analyses.

Within-subject Modelling

The timeseries data from these seed regions were extracted, detrended (using AFNI's 3dDetrend) to remove baseline scanner drift, transposed (using AFNI's 1dcat), upsampled to a TR of 0.25 alongside the stimulus onset times (using AFNI's '1dUpsample' for timeseries and AFNI's timing_tool.py for stimulus timings) in order to be deconvolved (using AFNI's '3dTfitter') with a gamma shaped hemodynamic response function (HRF). This then allows gPPI regressors to be obtained (using AFNI's '1deval') for the interaction between the seed region timeseries and the stimuli onsets of interest (see within-subject modelling in the neural activation methods subsection). These gPPI regressors were convolved with the HRF (using AFNI's 'waver') and down-sampled back to the original TR of 3.5 (using AFNI's '1dcat'). For each seed region, we then constructed a within-subject GLM using as regressors of interest the gPPI interaction terms. As nuisance regressors we included the HRF-convolved stimuli onset times, seed region timeseries and the motion parameters previously specified in the neural activation methods subsection. The resulting beta weight maps for the gPPI interaction terms were then subjected to the below group-level analyses.

Group-level ROI Analysis

In our group-level ROI analyses, we investigated functional connectivity between the left amygdala, right amygdala and sgACC seed regions with a dorsomedial frontal (dmFC) ROI defined by Nord et al. (2019) as well as the left amygdala and right amygdala seed regions and the ACC ROI from Garibbo et al., 2019. We compare the interaction between the seed regions (gPPI interaction terms) and ROIs during encoding under threat-of-shock to safety using the same two complementary approaches as for the ROI replication analysis (*see method section on neural activation*)

Group-level Whole-brain Analysis

We also ran group-level whole-brain functional connectivity analyses for the left amygdala, right amygdala and sgACC seed region (using AFNI's 3dMVM). The beta weights for the gPPI interaction terms of interest from the within-subject modelling are provided to '3dMVM' as within-subject regressors (using 'wsVars' option). We used a template MNI grey matter mask. The contrast of interest was defined as the gPPI interaction terms for threat-of-shock > safety during encoding. We accounted for family-wise errors using simulation-based cluster-correction (*see neural activation methods subsection on whole-brain analysis*) with a voxelwise-threshold of $p<0.001$ and bi-sided cluster-level significance threshold of $p<0.05$.

We found no significant results in our planned functional connectivity ROI analysis for encoding under threat-of-shock compared to safety between the dorsomedial frontal cortex (dmFC) ROI and left amygdala (t=0.765, df=91, p=0.446, $CI_{95\%}$=[-0,313, 0.706], $Mean$=0.196), right amygdala (t=0.217, df=91, p=0.828, $CI_{95\%}$=[-0,490, 0.611], $Mean$=0.06), or subgenual anterior cingulate cortex (sgACC) (t=-0.969, df=91, p=0.335, $CI_{95\%}$=[-0,838, 0.385], $Mean$=-0.272). Further, no clusters emerged for any of the seed regions within the dmFC ROI when using small volume correction. We also found no significant results in our planned functional connectivity ROI analysis for encoding under threat-of-shock compared to safety between the anterior cingulate (ACC) ROI and left amygdala (t=-1.033, df=91, p=0.304, $CI_{95\%}$=[-0.657, 0.208], $Mean$=-0.225), right amygdala (t=-0.075, df=91, p=0.941, $CI_{95\%}$=[-0.445, 0.412], $Mean$=-0.016). No clusters emerged for any of the seed regions within the ACC ROI when using small volume correction.

In the planned functional connectivity whole-brain analysis we also found no significant results for the left amygdala, right amygdala or sgACC seed region (voxel-wise threshold $p<0.001$, cluster-level significance threshold $p<0.05$).

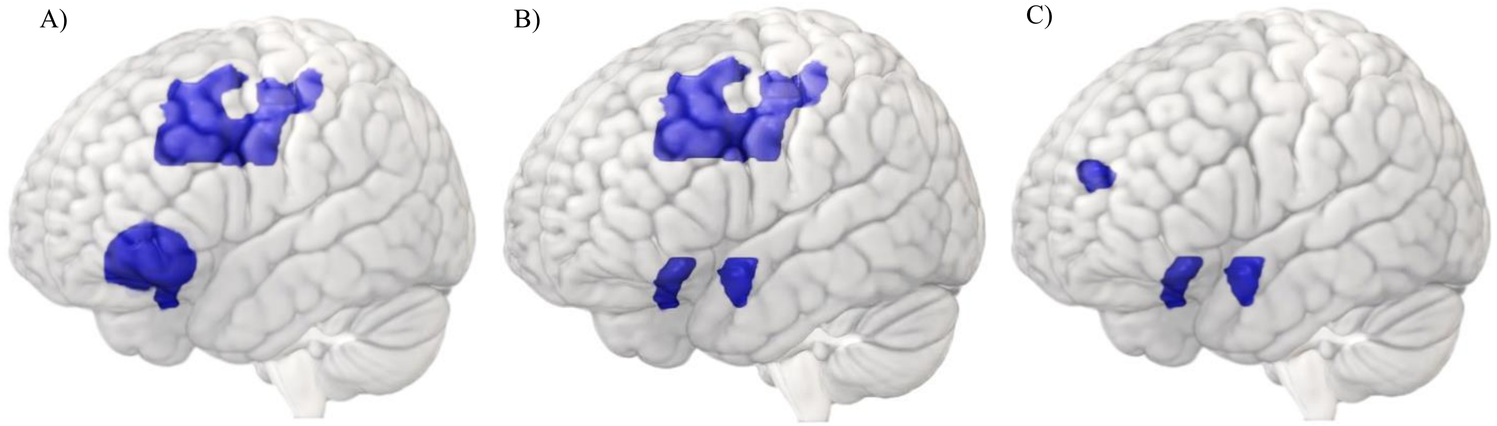

Supplementary Figure 8. Functional Connectivity Analysis *of the psychophysiological interaction during threat-of-shock compared to safe encoding between A) the sgACC seed region and the dmFC ROI, B) the left and right amygdala seed regions (note these were analysed separately) and the dmFC ROI and C) the left and right amygdala seed regions (analysed separately) and the ACC ROI from Garibbo et al. (2019), none of which revealed significant results.*

Extending our investigation with a psychophysiological connectivity analysis revealed no statistically significant changes in a translational amygdala-fronto (i.e., cingulate cortex and DMFC) circuitry associated with threat-of-shock during encoding. In general, psychophysiological interaction effects can be difficult to detect due to a lack statistical power (O'Reilly et al., 2012). This is because both the stimuli onset times and the seed region timeseries are included in the general linear model alongside the interaction term, which is a product of the two. Since this substantially reduces the variance that can be uniquely explained by the gPPI terms, the power to detect psychophysiological interaction effects is massively reduced and a high rate of false negatives should be expected. It is also worth noting that deconvolving the BOLD signal timeseries of seed regions to obtain the gPPI terms relies on making assumptions about the shape of the HRF, which we cannot verify. Given this was the first application of the gPPI approach to the threat-of-shock potentiated face recognition task, there was no prior work to directly guide our selection of seed and ROI regions. Our planned connectivity analyses were therefore limited to prior hypotheses based on other related paradigms, which might not be well suited to capture functional connectivity patterns underlying our task. For instance, previous work found increased dmPFC-amygdala

connectivity under threat-of-shock during the processing of fearful, but not happy, faces, suggesting that this circuitry may respond differentially to aversive face stimuli and not face salience in general (Robinson et al., 2012). Future connectivity analyses could take a more data-driven approach and investigate coupling between a broader range of brain regions. Based on our neural activation results, future neuroimaging analyses may also explore networks specifically related to internally directed attentional processing, such as the default mode or frontoparietal control network, and ask if these are preferentially recruited during the encoding stage (under threat-of-shock) in this or similar tasks.
