# Supplementary material for: Independent replications reveal anterior and posterior cingulate cortex activation underlying state anxiety-attenuated face encoding
Source: Commun Psychol. 2024 Aug 24;2:80. doi: 10.1038/s44271-024-00128-y (PMC11343718; doi:10.1038/s44271-024-00128-y)
Supplement: Supplementary file 3 — Reporting Summary [file 44271_2024_128_MOESM3_ESM.pdf]

nature portfolio

Corresponding author(s): Manuscript COMMSPSYCHOL-24-0022-T

Last updated by author(s): Feb 20, 2024

Reporting Summary

Nature Portfolio wishes to improve the reproducibility of the work that we publish. This form provides structure for consistency and transparency in reporting. For further information on Nature Portfolio policies, see our Editorial Policies and the Editorial Policy Checklist.

Statistics

For all statistical analyses, confirm that the following items are present in the figure legend, table legend, main text, or Methods section.

n/a	Confirmed	
☐	☒	The exact sample size (n) for each experimental group/condition, given as a discrete number and unit of measurement
☐	☒	A statement on whether measurements were taken from distinct samples or whether the same sample was measured repeatedly
☐	☒	The statistical test(s) used AND whether they are one- or two-sided<br>*Only common tests should be described solely by name; describe more complex techniques in the Methods section.*
☒	☐	A description of all covariates tested
☐	☒	A description of any assumptions or corrections, such as tests of normality and adjustment for multiple comparisons
☐	☒	A full description of the statistical parameters including central tendency (e.g. means) or other basic estimates (e.g. regression coefficient) AND variation (e.g. standard deviation) or associated estimates of uncertainty (e.g. confidence intervals)
☐	☒	For null hypothesis testing, the test statistic (e.g. F, t, r) with confidence intervals, effect sizes, degrees of freedom and P value noted<br>*Give P values as exact values whenever suitable.*
☒	☐	For Bayesian analysis, information on the choice of priors and Markov chain Monte Carlo settings
☒	☐	For hierarchical and complex designs, identification of the appropriate level for tests and full reporting of outcomes
☐	☒	Estimates of effect sizes (e.g. Cohen's d, Pearson's r), indicating how they were calculated

Our web collection on statistics for biologists contains articles on many of the points above.

Software and code

Policy information about availability of computer code

Data collection	Code in Matlab was used to run the task
Data analysis	R was used for behavioural data analysis, and AFNI for fMRI data analysis

For manuscripts utilizing custom algorithms or software that are central to the research but not yet described in published literature, software must be made available to editors and reviewers. We strongly encourage code deposition in a community repository (e.g. GitHub). See the Nature Portfolio guidelines for submitting code & software for further information.

Data

Policy information about availability of data

All manuscripts must include a data availability statement. This statement should provide the following information, where applicable:

- Accession codes, unique identifiers, or web links for publicly available datasets
- A description of any restrictions on data availability
- For clinical datasets or third party data, please ensure that the statement adheres to our policy

The fully anonymized and non-identifiable behavioural data that support the main findings of this study, including data from the current sample as well as previous studies analysed (Bolton & Robinson, 2017; Garibbo et al., 2019), are publicly available on OSF: https://osf.io/6952a/.

The group-level statistical maps for fMRI data that support the main findings of this study are publicly available on Neurovault: https://neurovault.org/

collections/16528/. Individual participant fMRI data are not openly available as they cannot be anonymised but may be made available from the corresponding author upon request and completion of a data sharing agreement.

Human research participants

Policy information about studies involving human research participants and Sex and Gender in Research.

Reporting on sex and gender	No sex- and gender-based analyses have been performed as there were no hyptheses regarding this. We report the age and sex/gender based on self-reported information provided by participants, but we do not report data on race/ethnicity in this study.
Population characteristics	During an initial screening, the Mini International Neuropsychiatric Interview (MINI; Sheehan et al., 1998) was administered to ensure no personal or family history of psychiatric disorders. Further exclusion criteria included: (i) general functional magnetic resonance imaging (fMRI) exclusions, (ii) general ill health, (iii) recent use of illicit drugs.
Recruitment	Participants were recruited using subject databases at University College London, Kings College London, the GLAD study (part of NIHR BioResource), MQ Participate and social media advertisements
Ethics oversight	UCL Research Ethics Committee (6198/002)

Note that full information on the approval of the study protocol must also be provided in the manuscript.

Field-specific reporting

Please select the one below that is the best fit for your research. If you are not sure, read the appropriate sections before making your selection.

☐ Life sciences ☒ Behavioural & social sciences ☐ Ecological, evolutionary & environmental sciences

For a reference copy of the document with all sections, see nature.com/documents/nr-reporting-summary-flat.pdf

Behavioural & social sciences study design

All studies must disclose on these points even when the disclosure is negative.

Study description	Quantitative
Research sample	Sample recruited from the (London based) general population, subject to self selection bias.<br>Gender: 71% female, 29% male, Age: Mean 24.22, SD 7.35, Range 18-60.
Sampling strategy	The sample was recurited using convenience sampling. The target sample size was based on a power calculation for a larger repeated-measures group comparison study, which required 45 subjects per arm to detect a medium effect size of 0.6 with 80% power (alpha 0.05, two-tailed between-subjects), as the current sample forms the non-symptomatic baseline group.
Data collection	A computer and MRI scanner were used for data collection. The researcher was not blinded to the experimental conditions, but their order was randomized.
Timing	Between December 2017 and May 2022.
Data exclusions	Of 98 participants 5 did not complete the task of interest so the final sample size for this study was N=93. From the original sample of 93, one participant had to be excluded from the bevahioural analysis because no responses were recorded, so the sample size for all behavioural data analyses was N=92. From the original sample of 93 (including the participant excluded from behavioural analysis), a different subject had to be excluded due to scanner artifacts so the final sample size for the fMRI data analysis was N=92.
Non-participation	An initial n=121 were recruited into the study. Of that 22 withrew after initial screening (non-response to baseline visit invitation) and 1 withrew due to MRI safety concerns, resulting in the n=98
Randomization	Participants were assigned to this group based on an initial screening using the Mini International Neuropsychiatric Interview (MINI; Sheehan et al., 1998) to ensure no personal or family history of psychiatric disorders. The experimental task involves a within-subjects design, so there was no allocation to experimental conditions.

Reporting for specific materials, systems and methods

We require information from authors about some types of materials, experimental systems and methods used in many studies. Here, indicate whether each material, system or method listed is relevant to your study. If you are not sure if a list item applies to your research, read the appropriate section before selecting a response.

Materials & experimental systems

n/a	Involved in the study
☒	☐ Antibodies
☒	☐ Eukaryotic cell lines
☒	☐ Palaeontology and archaeology
☒	☐ Animals and other organisms
☒	☐ Clinical data
☒	☐ Dual use research of concern

Methods

n/a	Involved in the study
☒	☐ ChIP-seq
☒	☐ Flow cytometry
☐	☒ MRI-based neuroimaging

Magnetic resonance imaging

Experimental design

Design type: Task-based block design

Design specifications: The task consisted of 4 blocks, each involving an encoding condition followed by a retrieval condition, alternating between a threat-of-shock (ToS) state and safe control state (see figure 1a and 1b). This constituted a 2 (safe vs threat) by 2 (encoding vs retrieval) design and resulted in the following combinations of encoding and retrieval respectively: safe-ToS, safe-safe, ToS-safe, ToS-ToS. Each block contained a different set of 36 face stimuli (i.e., 144 in total) from the Chicago Face Database (Ma et al., 2015), with equal ratios of male to female as well as happy, fearful and neutral faces. Block order was counterbalanced across participants to account for shock desensitization over time and stimuli order was randomized within blocks. First, during encoding, 18 face stimuli were sequentially presented for 0.5 seconds each, separated by a fixation cross with an inter-stimulus interval between 0.75 and 2 seconds (ISI). After a longer fixation interval, between 7.5 and 12.5 seconds, participants entered the retrieval phase. During retrieval, 36 face stimuli were presented sequentially for 0.5 seconds, half of which were previously presented during encoding and the other half unseen. Following another ISI, participants were presented with a response slide asking whether they had seen the face before. This was on screen either until they responded by pressing a button corresponding to Yes or No (counterbalanced), or until more than 2 seconds passed, in which case an incorrect response was automatically recorded. Before and after the task a 30 second fixation was presented to provide an additional baseline for fMRI contrasts.

Behavioral performance measures: Responses and Reaction time was recording via button presses

Acquisition

Imaging type(s): functional

Field strength: 1.5

Sequence & imaging parameters: A field map (T2*-weighted images: repetition time (TR) = 1170ms, echo time (TE1) = 10ms, TE2 = 14.76ms, field of view (FOV) = 64x64, voxel size = 3x3x2mm, slice thickness = 2mm, flip angle = 90°, 64 volumes) was obtained whilst participants completed the training block, EPI scans (T2*-weighted images: repetition time (TR) = 3500ms, echo time (TE) = 50ms, field of view (FOV) = 64x64, voxel size = 3mm, slices = 40, slice thickness = 2mm, flip angle = 90°, approximately 228 volumes) were collected during the testing block, and an MPRAGE (T1-weighted images: TR = 2730ms , TE = 3.57ms, FOV = 224x256, voxel size = 1mm, slice thickness = 1mm, flip angle = 7°, 176 volumes) at the end of the session.

Area of acquisition: Whole-brain

Diffusion MRI: ☐ Used ☒ Not used

Preprocessing

Preprocessing software: All fMRI data was pre-processed using the open source fMRIPrep pipeline version 20.2.7 (for full details see https://fmriprep.org/en/20.2.7/workflows.html). In addition to fMRIPrep, we spatially smoothed data to 6mm FWHM, constrained within a MNI template grey matter mask (AFNI's '3dBlurToFWHM') and scaling of the timeseries in each voxel to a mean of 100 (AFNI's '3dTstat', '3dcalc'). Before data analysis, the first four volumes were discarded to allow the magnetic field to stabilize.

Normalization: Spatial normalization to standard MNI152NLin2009cAsym space ('ANTs')

Normalization template: MNI152NLin2009cAsym

Noise and artifact removal: As nuisance regressors in all within-subject GLMs we included movement-correction parameters (pitch, roll, yaw, z, y, z and derivatives of each motion type) as well as a parameter controlling for the time of shock delivery and presentation time of

the safe/threat warnings. To further control for motion artifacts, we censored volumes with framewise displacement exceeding 1.3 mm and excluded individuals with more than 20% of volumes requiring censoring (none in this sample).

Volume censoring

In Afni we censored volumes with framewise displacement exceeding 1.3 mm and excluded individuals with more than 20% of volumes requiring censoring

Statistical modeling & inference

Model type and settings

For the within-subject modelling we constructed general linear models (GLMs). With the regressors of interest we accounted for both preceding and subsequent state, threat-of-shock (TH) or safety (SF), in our contrasts of encoding and retrieval by including the face stimuli onset times for all combinations of state at encoding (encoding face onsets: THTH, THSF, SFTH, SFSF) and retrieval (retrieval face onsets: THTH, THSF, SFTH, SFSF). Then we also included the contrasts for TH encoding > SF encoding and TH retrieval > SF retrieval. These were convolved with the hemodynamic response function (approximated by a gamma function) using '3dDeconvolve' in AFNI.

Effect(s) tested

For whole-brain and ROI analyses in the ACC we used the following contrast of interest: threat-of-shock > safety, during encoding and retrieval separately. For ROI in the hippocampus we used the following contrast of interest: state congruent retrieval (threat-of-shock/safety at encoding followed by threat-of shock/safety at retrieval) > incongruent retrieval (threat-of-shock/safety at encoding followed by safety/threat-of shock at retrieval).

Specify type of analysis: ☐ Whole brain ☒ ROI-based ☐ Both

Anatomical location(s): ACC, Hippocampus

Statistic type for inference
(See Eklund et al. 2016)

Cluster-wise Inference.

Correction

We used simulation-based cluster-correction, using AFNI's '3dClustSim' to estimate, based on simulations of false positive noise clusters (derived by '3dFWHMx' from spatial autocorrelation estimates of 3dMVM's group-level model residuals), the minimum required cluster size for a specified voxelwise-threshold of $p<0.001$ and significance threshold of $p<0.05$. We used bi-sided thresholding, whereby positive and negative values above the threshold are clustered separately, and AFNI's NN level 2, whereby clusters are defined when faces or edges touch. The small volume correction approach allows for effects to emerge when they are driven by smaller clusters within the ROI, which can be especially important for large ROIs, or when both activation and deactivation occur within an ROI. For these analyses we cannot report exact values for the t-statistic, p-value and confidence interval but only the minimum cluster size required.

Models & analysis

n/a	Involved in the study
☒	☐ Functional and/or effective connectivity
☒	☐ Graph analysis
☒	☐ Multivariate modeling or predictive analysis
